# Mice Placental ECM Components May Provide A Three-Dimensional Placental Microenvironment

**DOI:** 10.3390/bioengineering10010016

**Published:** 2022-12-22

**Authors:** Rodrigo da Silva Nunes Barreto, Ana Claudia Oliveira Carreira, Mônica Duarte da Silva, Leticia Alves Fernandes, Rafaela Rodrigues Ribeiro, Gustavo Henrique Doná Rodrigues Almeida, Bruna Tassia dos Santos Pantoja, Milton Yutaka Nishiyama Junior, Maria Angelica Miglino

**Affiliations:** 1 Department of Surgery, School of Veterinary Medicine and Animal Science, University of São Paulo Cidade Universitária, Butantã CEP 05508-270, Brazil; 2 Center for Natural and Human Sciences (CCNH), Federal University of ABC, Santo André CEP 09210-580, Brazil; 3 Laboratory for Applied Toxinology, CeTIS, Butantan Institute, São Paulo CEP 05503-900, Brazil

**Keywords:** animal models, materno-fetal interface, placental ECM proteomics

## Abstract

Bioethical limitations impair deeper studies in human placental physiology, then most studies use human term placentas or murine models. To overcome these challenges, new models have been proposed to mimetize the placental three-dimensional microenvironment. The placental extracellular matrix plays an essential role in several processes, being a part of the establishment of materno-fetal interaction. Regarding these aspects, this study aimed to investigate term mice placental ECM components, highlighting its collagenous and non-collagenous content, and proposing a potential three-dimensional model to mimetize the placental microenvironment. For that, 18.5-day-old mice placenta, both control and decellularized (*n* = 3 per group) were analyzed on Orbitrap Fusion Lumos spectrometer (ThermoScientific) and LFQ intensity generated on MaxQuant software. Proteomic analysis identified 2317 proteins. Using ECM and cell junction-related ontologies, 118 (5.1%) proteins were filtered. Control and decellularized conditions had no significant differential expression on 76 (64.4%) ECM and cell junction-related proteins. Enriched ontologies in the cellular component domain were related to cell junction, collagen and lipoprotein particles, biological process domain, cell adhesion, vasculature, proteolysis, ECM organization, and molecular function. Enriched pathways were clustered in cell adhesion and invasion, and labyrinthine vasculature regulation. These preserved ECM proteins are responsible for tissue stiffness and could support cell anchoring, modeling a three-dimensional structure that may allow placental microenvironment reconstruction.

## 1. Introduction

The placenta plays an essential role in conceptus maintenance in the uterine environment, supplying oxygen, and nutrients and protecting it against harmful exogenous agents present in maternal blood flow [1]. The materno-fetal interaction has been investigated to understand the appropriate conditions for embryo and fetal development [2]. Early complications during embryo implantation impacts directly on placental development leading to gestational losses [3].

Several studies attempted to comprehend the physiological aspects of human placentation, most of them using explants and derived progenitor cells from unsuccessful or term pregnancies [4]. Mice placenta has been considered a classic placental model for several approaches due to their similar hemochorial placenta, including the development of transgenic animals for functional and molecular in vivo and in vitro studies [5,6,7,8,9]. In addition, mice’s placenta advantages include easy manipulation, small size, short generation time, and genetic homogeneity, followed by several morphological and functional similarities [6].

Despite these advantages and similarities, new three-dimensional and more versatile models to better mimetize the human placental microenvironment. For this purpose, tissue bioengineering strategies, such as placental fragments reconstruction, and applying cells and biomaterials are being explored [10]. However, information about trophoblast cell culture in biological scaffolds is scarce.

Placental ECM not only contributes to structural support but also regulates cellular signaling-modulating processes such as proliferation and motility [11]. These models can also be applied for physiological and pharmacological assays, such as experimental vertical infections, toxic molecules investigation, and drug therapies [12]. However, the use of models that do not properly mimic the placenta environment leads to unreliable knowledge, which requires alternatives to characterize functional, structural, and molecular aspects of the placenta [13,14,15,16]. To overcome this problem, placental organoids from three-dimensional (3D) microenvironment culture, simulating the materno-fetal interactions, have been considered a reliable model to study molecular effects on the placenta [17,18,19,20,21]. Moreover, 3D cultures display better migration and invasion profiles, and resistance to viral and microbial infections [4,20,22,23,24].

Human and murine trophoblastic cell populations present a functional dynamism during placental development [25]. In mice, placental hormonal activity is restricted to the outer trophoblast layer (syncytiotrophoblast), while transport and barrier functions are majorly performed by the two inner layers (trophoblast giant cells and spongiotrophoblast) [6]. Mice’s placental transcriptional and proteomic profile during each embryonic stage elucidate several mechanisms in cell interactions, including its organization and maturation [26,27,28]. Differently, human placenta physiology cannot be precisely understood only by samples derived from term and unsuccessful pregnancies [4].

A suitable in vitro placental model is highly influenced by ECM tridimensional structure, where its architectural stiffness is essential [29]. As a transient organ, the placental ECM presents a unique plasticity profile due to short-time development and loss of function for placental release [30]. To produce a mouse placenta ECM as an innovative biomaterial to support cells growth and differentiation [31] is essential to know and maintain its composition profile based on structural proteins (collagens and elastin), adhesion glycoproteins (fibronectin, laminin, tenascins, and vitronectin), glycosaminoglycans (hyaluronic acid), proteoglycans (versican, syndecan, glypican, and perlecan), matricellular proteins (osteonectin, thrombospondin, tenascin, osteopontin) and metalloproteinases (MMP-2 and MMP-9) [32]. Thus, this investigation considered and described the possibility of a new mice placental model, based on late pregnancy three-dimensional extracellular matrix microenvironment.

## 2. Material and Methods

### 2.1. Decellularization Process

Placenta from E18.5 mice (N = 03, in each control and decellularized group) were obtained according to the protocol established by Barreto et al. [31]. The decellularization process was carried out using crescent concentrations of SDS (0.01%, 0.1%, and 1%), and 1% Triton X-100. This study was approved by the Ethical committee on the use of animals (No. 5669271015) from the School of Veterinary Medicine and Animal Science of the University of Sao Paulo.

### 2.2. Mass Spectrometry Samples

Control (C1-C3) and decellularized (D1-D3) mice placenta biological replicates (*n* = 3) were processed accordingly established by Hedrick et al. [33], Matias et al. [34] and Barreto et al. [35]. Briefly, samples were homogenized with 1 mL (100 mM) ammonium bicarbonate solution (ABC); precipitated with acetone (1:4) at −20 °C for 16 h; reduced with 8 M urea and 10 mM DTT for 2 h at 37 °C; alkylated with 200 µM Iodoacetamide, digested with 0.1 µg/µL trypsin under a barocycler; and purified in C18 columns (300 Å, #SMM SS18V, The Nest Group, Inc., Ipswich, MA, USA). The data generated by Orbitrap Fusion Lumos spectrometer (Thermo Scientific) were deposited in the Mendeley Data database in different datasets for control (doi:10.17632/yg5phbft32.1, accessed on 8 October, 2022) and decellularized (doi:10.17632/wkdsh9kf9t.1 accessed 8 October 2022) groups. The Orbitrap Fusion Lumos spectrometer has maximized instrument performance and flexibility allowing more confident, precise, and sensible detection even with a low sample number [36]. In addition, this instrument associated with a precise pipeline and batch analysis increases the data trustworthiness [37].

### 2.3. Data Collection and Bioinformatic Analysis

There was used the Label-Free Quantification MaxLFQ algorithm, a semi-quantitative protein analysis, from MaxQuant software (version v1.6.10.43) [38] with an FDR rate of 1% to compare the relative abundance of proteins based on the mice proteins database from Uniprot/Swissprot, for each control and decellularized mice placenta samples and respective replicates. Proteins identified in the contaminant database and the decoy database were removed. For the criterion for protein identification, it was considered that only peptides identified with the posterior error probability (PEP) ≤ 0.01 in at least one biological replicate, and the occurrence of at least one unique peptide. We considered the intensity values of the LFQ that are normalized by the Maxquant software based on the sum of the intensity of all peptides of all identified proteins. LFQ for each protein was considered when the intensity data were present in at least two out of three replicates. Further, the protein abundance and log2 fold change (log2(FC)) for each group were calculated based on the average quantification of biological replicates, identifying the significantly quantified proteins with a Fold Change higher than 1.5 (|log2(FC)| ≥ 0.585). Following there were conducted the ANOVA and T-test (*p* < 0.05) statistical tests to determine the protein *p*-values, using the Microsoft Excel software (Matias et al. [34]; Barreto et al. [35]). In addition, proteins that had a zero value in two of the three conditions were analyzed separately. The data quality was checked by means of correction graphs and principal component analysis. After, we selected ontologies related to ECM and cell junctions (Supplemental Table S1) on the cell component domain. Then, the filtered protein list was used for principal component analysis (PCA), which was applied to find which combinations of the differentially quantified proteins with a fold change higher than 1.5. The PCA analysis was performed using the R-statistics package FactoMineR [39] and Factoextra (http://www.sthda.com/english/rpkgs/factoextra, accessed on 8 October 2022) for graphical visualization. False Discovery Rate adjustment was calculated by the Bonferroni method. Enrichment analysis and functional classification for gene ontology terms (“enrichGO” function from R package clusterProfiler) [40]; proteins enrichment in KEGG pathways (“enrichKEGG” function from clusterProfiler package and Pathviews package from R) [41]; and biological network interactions of proteins (NetworkAnalyst [42]). The Clustering analysis was performed using R statistical software version 3.6.3 (http://www.R-project.org, accessed 8 October 2022). The set of protein dissimilarities were computed using the “Euclidean” distance with the function “dist” to the hierarchical clustering based on the package and function “hclust”. There was employed the agglomerative method with “ward.D2”. All bioinformatics analysis was performed as described by Matias et al. [34] and Barreto et al. [35].

## 3. Results

**Figure 1:**
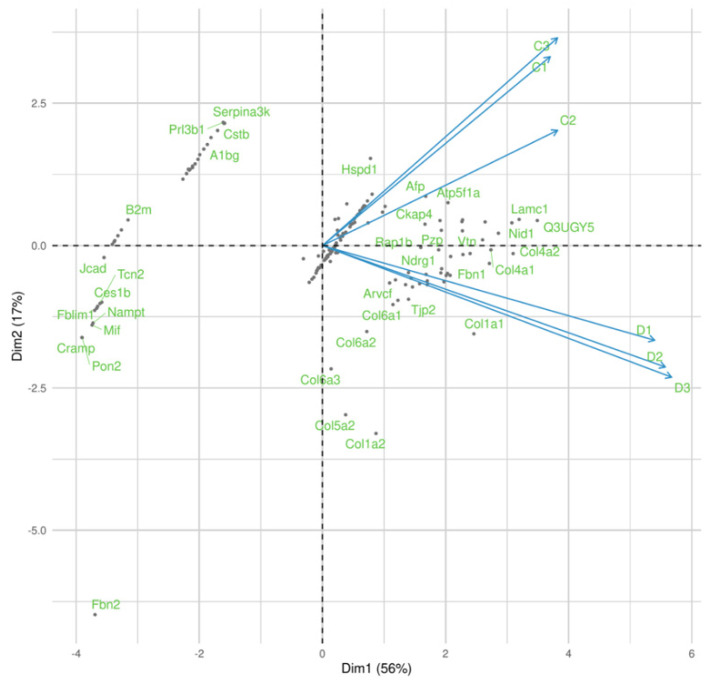
Principal component analysis (PCA, Spearman correlation) plot from control (C1–C3) and decellularized (D1–D3) mice placenta. Those graphs show the consistency of each sample with their condition (control and decellularized).

ECM proteomic profiles from control and decellularized mice placenta were analyzed to determine if the remaining proteins could provide a tridimensional cell culture microenvironment. Principal component analysis (PCA, Spearman correlation) initially displayed that control and decellularized samples were spaced and clustered by biological replicates in separated quadrants, consistent with their respective condition (Figure 1). On PCA, decellularized quadrant enriched several collagen types, whereas the control quadrant enriched proteins related to cell adhesion (i.e., Vtn, Nid1, Lamc, and Ckap4).

The MaxQuant assembling of mass spectrometry detected peptides, generating a list of 2317 proteins and 118 (5.1%) proteins resulting from ECM and cell junction-related ontologies filtering. From those proteins, using fold change (higher than 1.5) and *p*-value (0.05), 40 (33.9%) proteins were overregulated in control mice placenta, whereas 76 (64.4%) had no significant differential expression between control and decellularized conditions. However, 2 (1.7%) of those were upregulated in decellularized mice placenta (Supplemental Table S2). From those, there were 76 proteins with no significant differential expression, several ECM proteins were preserved: collagens (Col1a1, Col4a1, Col4a2, Col6a1, Col6a2, Col6a3, Col14a1, Col18a1); laminins (Lama1, Lama4, Lama5, Lamb2, Lamc1); Fibrillin (Fbn1); Fibronectin (Fn1); glycoproteins [Bgn, Hspg2, Nid1] and cell junction-related proteins [Arvcf, Coch, Emilin1, Esam, Igf2bp1, Itga6, Lad1, Lims1, Mpp5, Parvb, Pak2, Pdlim1, Pdlim2, Pkp2, Plg, Pvr, Serpine1, Sorbs1, Tjp1, Tjp2, Utrn, Vasp, Vtn]. In addition, two collagens (Col1a2 and Col5a2) were upregulated in the decellularized placenta. Among the upregulated proteins in the control condition, some were related to ECM modulation (Htra1, Htra3, Plod3, Sparc), or cell adhesion (F11r, Itga2b, Itga5, Itgav, Lgals3bp).

In total, 25 ontologies of the cellular component domain were closely related, forming a unique interaction, with some inferred relationships (dotted lines) (Supplemental Figure S1). On the biological process domain, 63 ontologies were closely interacted (Supplemental Figure S2), while on the molecular function domain only 21 were interconnected (Supplemental Figure S3).

Inside the 30 more relevant ontologies from each of the three domains, we found 11 (36.7%) ontologies related to cell junction in the cellular component domain, six (20%) related to collagen, and another six (20%) related to lipoprotein particles (Supplemental Figure S4). In the biological process domain, 10 (33.3%) ontologies were related to cell adhesion, six (20%) to the vasculature, five (16.7%) to proteolysis, and three (10%) to ECM organization (Supplemental Figure S5). Finally, among the molecular function domain, 16 (53.3%) were related to protein binding, 11 (36.7%) to protein activity, and three (10%) with ECM resistance (Supplemental Figure S6).

**Figure 2:**
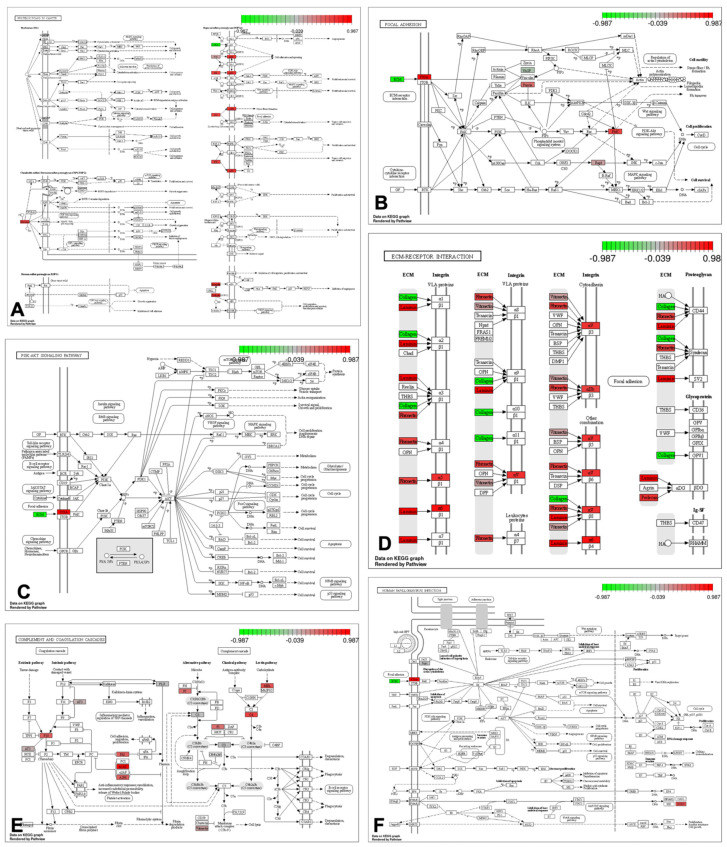
Six most relevant KEGG pathways enriched from detected filtered proteins on control versus decellularized mice placenta. The upregulated proteins are in red color and the downregulated ones in green color. (**A**). Proteoglycans in cancer pathway. (**B**) Focal adhesion pathway. (**C**) PI3K-AKT signaling pathway. (**D**) ECM-receptor interaction. (**E**) Complement and coagulation cascades pathway. (**F**) Human papillomavirus infection pathway.

**Figure 3:**
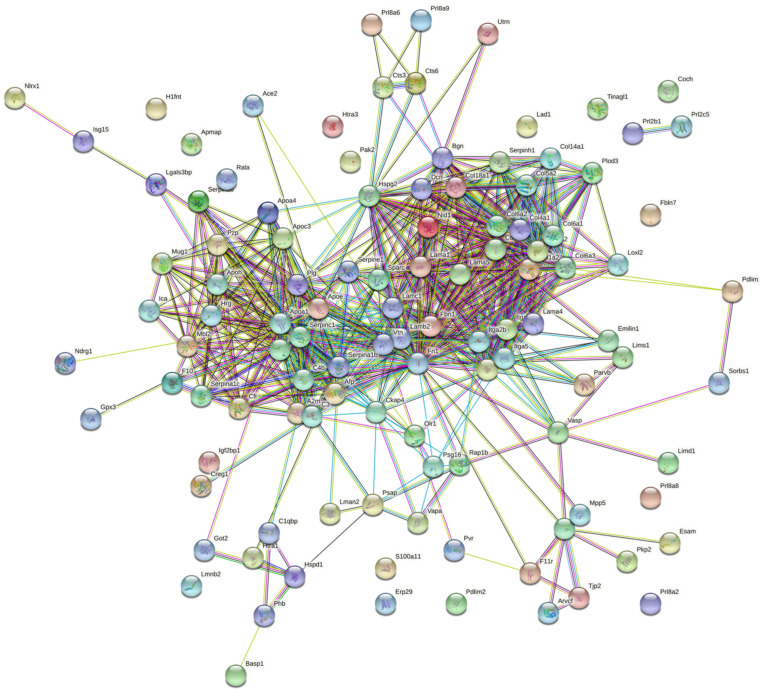
Protein-protein interactions of the 118 proteins on control and decellularized mice placenta, according to the STRING database. Most proteins were connected in one big cluster, suggesting a strong functional relationship between them. The connecting nodes indicate protein-protein interactions with medium interaction confidence of 0.4.

The major pathways which enriched more proteins were: Focal adhesion (30.5%), ECM-receptor interaction (28.0%), Human papillomavirus infection (28.0%), PI3K-Akt signaling pathway (26.8%), Complement and coagulation cascades (19.5%) and Proteoglycans in cancer (14.6%) (Figure 2). Together those pathways enriched several collagen types and integrins. Other pathways were also enriched on several proteins (Supplemental Figure S7). The constructed String DB interactome assembled 80 (68%) proteins in just one cluster (Figure 3), showing the proteins’ major amounts are interconnected and have interacted function.

## 4. Discussion

This study described the ECM-related protein profile on late gestation mice placenta after the decellularization process to verify if the derived scaffold was suitable to provide a tridimensional microenvironment model for cell culture and bioengineering.

Several proteins were kept after the decellularization process, demonstrated by similar detection in control and decellularized tissues. Within those proteins, the different collagen types observed are involved in fibril-forming (Col1a1), basement membrane (Col4a1 and Col4a2), beaded filament-forming (Col6a1, Col6a2a, and Col6a3), anchoring fibril-forming (Col6a1, Col6a2a, Col6a3, and Col14a1), and multiplexing (Col18a1) collagens [43]. The collagen type presence on decellularized placenta attests to the tridimensional architecture preservation, since this architecture is assembled by collagen fibers, where the thicker ones (60–330 nm) are supportive, and the thinner ones (15–30 nm) complement the ECM lattice structure, keeping labyrinthine capillary net and other structures [31,44]. Furthermore, different collagen fibers stabilize the structure by anchoring themselves with other ECM molecules and neighbor cells [45].

Other non-collagen proteins that also present structural and adhesive functions, such as laminins (Lama1, Lama4, Lama5, Lamb2, Lamc1), fibrillin (Fbn1), and fibronectin (Fn1), were preserved as well. These proteins usually have binding domains to several collagen types, adding strength to the tridimensional structure maintenance [46]. Results related to the ECM architecture and ultrastructural organization after decellularization were already shown in mice and other rodents [31,44], bovine [35,47], and canine [48,49], which described the vascular architecture maintenance, and basement membrane proteins preservation. In addition, laminin, fibronectin, and vitronectin, together, interact with integrin receptors of trophoblastic cells, promoting their adhesion [50,51]. Integrin spatial distribution is variable in different placental compartments, like villous and extravillous trophoblasts in humans [52] and labyrinth, junctional zone, and decidua in mice [53]. Moreover, trophoblast cell lines migration and invasion depend on integrins, which are transmembrane glycoprotein receptors that regulate cell differentiation, motility, and adhesion by cytoskeletal reorganization [54,55,56]. Altogether, those preserved collagens and non-collagenous proteins are enough to support several phases of tissue reconstruction, providing the basic microstructure for adhesion, migration, and cell differentiation [48,57].

From the cellular component domain, the ontologies related to cell junction, collagen, and lipoprotein particles were the most enriched ones. These collagen types bind to domains of several adhesive and transmembrane proteins, attaching the cells to each other, to the basement membrane, or to ECM [46]. Cell junction and collagen ontologies are related to each other, and their proteins were maintained in decellularized mice placenta. Placental lipoprotein particle ontology is also essential for syncytiotrophoblast hormonal metabolism, as well as for high fetal requirements [58].

From the biological process domain, the enriched ontologies were related to vasculature, cell adhesion, proteolysis, and ECM organization. For vasculature modulation, such as ECM organization, the microenvironment modulation is dependent on the proteolysis, by hydrolytic proteins, to degrade the natural ECM structure, and control ECM deposition [45]. Furthermore, one of the control mechanisms for cell adhesion and detachment is the proteolysis of adhesive proteins, which is responsible for binding the cell membrane to the ECM structure [59,60]. Additionally, in the molecular function domain, the protein binding, protein activity, and ECM resistance ontologies were enriched. These three ontologies are closely related, because the ECM resistance is more dependent on their protein structural arrangement, instead of protein amount [61,62].

From the enriched pathways, we could cluster them in cell adhesion and invasion, and labyrinthine vasculature regulation for placental nutrition. The focal adhesion pathway was the one with more proteins enriched, being closely related to key signaling for cell adhesion or detachment. Focal adhesion is a multi-protein complex structure on the cell membrane that anchors the cytoskeleton directly to ECM, giving the ability for the cell to respond to chemical or physical changes [63]. ECM-receptor interaction pathway mediates the direct or indirect interaction between ECM and transmembrane molecules (majorly integrins and proteoglycans), to control several cell functions and invasiveness [64]. Proteoglycans in the cancer pathway play an important role in cellular adhesion and invasion and control proteoglycan location and function through microenvironment enzyme alterations [65]. The human papillomavirus infection pathway in the placental microenvironment can be related to increased cell proliferation and p53 signaling inhibition. Likewise, the PI3K-Akt signaling pathway regulates trophoblast cell proliferation by decreasing apoptosis [66,67]. Complement and coagulation cascades pathway are related to support unclothed blood in the labyrinthine blood sinus to maintain syncytiotrophoblast nutrition and support hypercoagulation during labor [68,69].

The ECM biology supports placental physiology, and any placental dysfunctions rapidly lead to ECM modification in structure and/or composition, such as in preeclampsia and intrauterine growth restriction [70,71,72,73], hypoxia [74], and cloned pregnancies [35,75]. Even in normal placentation, the placental ECM is plastic and intensely modulated due to decidualization, placental development, and fetal requirement [45].

Furthermore, the placental ECM protein content is a key to in vitro placental modeling [76]. Decellularized placental ECM has a large potential to be used for modeling materno-fetal interface due to several difficulties in conducting in vitro experiments using primary placental cells and chorionic villous explants [77]. Besides ECM composition, ECM stiffness also influences cell physiology, which can range from 0.2 kPa in the brain to 10^6^ kPa in bone. Generally, the substrates used in cell culture have a stiffness different from the placenta tissues and directly influence placental cell survival [29]. For example, Matrigel^®^ has a stiffness of 331 Pa, whereas decidua basalis and parietalis have 1250 and 171 Pa, respectively [29]. The placental ability for materno-fetal circulation gas exchange [78], and their complex vascular network [79] can be translated to lung modeling [80]. In addition, the placenta can be approached for clinical translation, optimizing in vitro barrier models for vertical transmission studies, and elucidating the effect of harmful molecules and pharmaceutical therapies.

Moreover, the ECM can influence normal and/or abnormal cell progression [81], such as in tumor progression and metastasis. However, ECM structure and stiffness can be altered by tumoral development (Barreto, unpublished data). On the other hand, bronchial asthmatic ECM received smooth muscle cells and they recellularized the bronchial scaffold, showing success [82]. Another example refers to ECM-derived hydrogel’s positive effects on pulmonary fibrosis treatment [83]. However, several in vitro models do not perfectly mimetize a species-specific placental environment [84,85]. Altogether, herein the detected ECM proteins, ontologies, and pathways support the idea that the decellularized mice placenta preserve a stable tridimensional microenvironment for materno-fetal in vitro modeling to reach multiple approaches on placental biology.

## Data Availability

All relevant data is available within the manuscript.

## Supplementary Materials

The following supporting information can be downloaded at: https://www.mdpi.com/article/10.3390/bioengineering10010016/s1, Please see the Supplementary Figures S1–S7 and Tables S1 and S2.
