# Supplementary material for: Mice Placental ECM Components May Provide A Three-Dimensional Placental Microenvironment
Source: Bioengineering (Basel). 2022 Dec 22;10(1):16. doi: 10.3390/bioengineering10010016 (PMC9855196; doi:10.3390/bioengineering10010016)
Supplement: Supplementary file 1 [file bioengineering-10-00016-s001.zip › bioengineering-1998463-Supplentary Tables.pdf]

Supplemental Table S1 – Component cellular ontologies used for protein filtering

Ontology	Term
adherens junction	GO:0005912
basement membrane	GO:0005604
bicellular tight junction	GO:0005923
cell junction	GO:0030054
cell surface	GO:0009986
cell-cell adherens junction	GO:0005913
cell-cell junction	GO:0005911
collagen trimer	GO:0005581
collagen type I trimer	GO:0005584
collagen type III trimer	GO:0005586
collagen type IV trimer	GO:0005587
collagen type V trimer	GO:0005588
collagen-containing extracellular matrix	GO:0062023
extracellular matrix	GO:0031012
extracellular membrane-bounded organelle	GO:0065010
extracellular region	GO:0005576
extracellular space	GO:0005615
focal adhesion	GO:0005925
integrin complex	GO:0008305
lamin filament	GO:0005638
laminin complex	GO:0043256
laminin-3 complex	GO:0005608

Supplemental Table S2 – Fold change (FC) of extracellular matrix and cell junctions-filtered proteins detected in mice placenta before (C) and after (D) decellularization process.

Protein ID	Gene names	Protein names	log2 (FC = C/D)	P-Value
**Differential expression in decellularized condition**				
Q3TX57	Col1a2	Fibrillar collagen NC1 domain-containing protein	-0,9120	0,00000
Q3U962	Col5a2	Collagen alpha-2(V) chain	-0,7337	0,00000
**No significant differential expression**				
P11087	Col1a1	Collagen alpha-1(I) chain	-0,4878	0,00069
Q02788	Col6a2	Collagen alpha-2(VI) chain	-0,2325	0,15653
J3QQ16	Col6a3	Collagen; type VI; alpha 3	-0,1483	0,30977
Q61554	Fbn1	Fibrillin-1	-0,1320	0,08950
B2RQQ8	Col4a2	Collagen; type IV; alpha 2	-0,1298	0,03324
Q04857	Col6a1	Collagen alpha-1(VI) chain	-0,1255	0,33424
A0A3B2WBH9	Tjp2	Tight junction protein ZO-2	-0,1096	0,01870
Q9CQ73	Pkp2	Plakophilin 2	-0,1028	0,00945
Q99K41	Emilin1	Elastin microfibril interface-located protein 1	-0,0815	0,21093
P98203	Arvcf	Armadillo repeat protein deleted in velo-cardio-facial syndrome homolog	-0,0793	0,00039
Q8R1F1	Niban2	Protein Niban 2	-0,0702	0,04113
E9Q6R7	Utrn	Utrophin	-0,0655	0,01708
Q8R1G6	Pdlim2	PDZ and LIM domain protein 2	-0,0635	0,07971
P02463	Col4a1	Collagen alpha-1(IV) chain	-0,0534	0,45584
B9EHJ3	Tjp1	Tight junction protein ZO-1	-0,0522	0,01688
O70400	Pdlim1	PDZ and LIM domain protein 1	-0,0483	0,14064
Q3U102	Esam	Endothelial cell-selective adhesion molecule	-0,0450	0,05881
P70460	Vasp	Vasodilator-stimulated phosphoprotein	-0,0430	0,06365
Q9QXD8	Limd1	LIM domain-containing protein 1	-0,0367	0,26953
P06728	Apoa4	Apolipoprotein A-IV	-0,0333	0,20471
Q62417	Sorbs1	Sorbin and SH3 domain-containing protein 1 (Ponsin)	-0,0207	0,18566
Q3V1T9	Plg	Plasminogen	-0,0140	0,72921
Q3UGY5	Fn1	Fibronectin	-0,0128	0,46745
B2RRY4	Mpp5	Membrane protein; palmitoylated 5 (MAGUK p55 subfamily member 5)	-0,0105	0,79666
O88477	Igf2bp1	Insulin-like growth factor 2 mRNA-binding protein 1	-0,0046	0,69448
Q3V2G1	Apoa1	Apolipoprotein A-I	-0,0002	0,99304

Q3TNY9	Bgn	Biglycan	0,0053	0,92354
A8CVP4	Lims1	LIM and senescent cell antigen-like-containing domain protein	0,0115	0,64633
Q8CIN4	Pak2	Serine/threonine-protein kinase PAK 2	0,0121	0,52434
Q6GTX3	Apoe	Apolipoprotein E	0,0134	0,31517
E9QPX1	Col18a1	Collagen alpha-1(XVIII) chain	0,0147	0,20616
Q61001	Lama5	Laminin subunit alpha-5	0,0191	0,51286
Q61292	Lamb2	Laminin subunit beta-2	0,0207	0,64538
F8VQJ3	Lamc1	Laminin subunit gamma-1	0,0302	1,00000
P10493	Nid1	Nidogen-1 (Entactin)	0,0321	0,34033
B9EHM9	Prl2b1	Growth hormone d12 (Prolactin family 2; subfamily b; member 1)	0,0356	0,49976
P01027	C3	Complement C3	0,0370	0,12332
Q543J5	Serpinc1	Antithrombin	0,0441	0,35770
A0A0M6L0I6	Prl8a8	Growth hormone d14	0,0459	0,48358
Q9WV55	Vapa	Vesicle-associated membrane protein-associated protein A	0,0638	0,01683
P50543	S100a11	Protein S100-A11	0,0714	0,16108
Q91XV3	Basp1	Brain acid soluble protein 1	0,0781	0,00310
A0A087WSN6	Fn1	Fibronectin	0,0827	0,03372
B2RXW7	C4b	Complement component 4B	0,0852	0,01979
Q545R3	Ndrg1	N-myc downstream regulated gene 1	0,0981	0,02987
P19137	Lama1	Laminin subunit alpha-1	0,1110	0,12259
Q3UEK9	Ahsg	Alpha-2-HS-glycoprotein	0,1139	0,00301
P29788	Vtn	Vitronectin	0,1230	0,02721
Q05793	Hspg2	Basement membrane-specific heparan sulfate proteoglycan core protein	0,1305	0,04178
Q52L50	Rap1b	RAS related protein 1b	0,1625	0,01349
Q99JR5	Tinagl1	Tubulointerstitial nephritis antigen-like	0,1885	0,01666
Q3TWG9	Serpinh1	SERPIN domain-containing protein	0,1903	0,00669
P97927	Lama4	Laminin subunit alpha-4	0,2048	0,00373
Q61838	Pzp	Pregnancy zone protein	0,2307	0,00768
Q01339	Apoh	Beta-2-glycoprotein 1	0,2527	0,03459
Q8CC06	Itga6	Integrin_alpha2 domain-containing protein	0,2619	0,01181
A0A0R4J0X5	Serpina1c	Alpha-1-antitrypsin 1-3	0,2731	0,00717
Q8BMK4	Ckap4	Cytoskeleton-associated protein 4	0,3149	0,00048
Q03265	Atp5f1a	ATP synthase subunit alpha; mitochondrial	0,3473	0,00082
Q61129	Cfi	Complement factor I	0,4000	0,00001
P63038	Hspd1	60 kDa heat shock protein; mitochondrial	0,4016	0,00133
Q3UGT9	Parvb	Parvin; beta	0,4189	0,00002

D3YY36	Ica	Inhibitor of carbonic anhydrase	0,4321	0,00017
Q9DAZ8	Cts3	Uncharacterized protein	0,4431	0,00000
P58022	Loxl2	Lysyl oxidase homolog 2	0,4433	0,00003
B7ZNH7	Col14a1	Collagen; type XIV; alpha 1	0,4457	0,00002
D0ESZ6	Serpine1	Serine or cysteine peptidase inhibitor clade E member 1 (Fragment)	0,4492	0,00053
Q9JLV9	Prl2c5	Prolactin-2C5 (Mitogen-regulated protein 4)	0,4528	0,00057
Q9EQ09	Olr1	Oxidized low-density lipoprotein receptor 1	0,4636	0,00007
P46412	Gpx3	Glutathione peroxidase 3	0,4712	0,00006
Q6GQT1	A2m	Alpha-2-macroglobulin-P	0,4768	0,00001
Q3U0Z1	Coch	Cochlin	0,4894	0,00009
Q8BK56	Afp	Alpha-fetoprotein	0,4897	0,00007
Q8R3D5	Lad1	Ladinin-1	0,4944	0,00000
P63321	Rala	Ras-related protein Ral-A	0,4970	0,00001
Q91WP1	Pvr	Cd155 (Poliovirus receptor)	0,4978	0,00006
**Differential expression in control condition**				
O88792	F11r	Junctional adhesion molecule A (JAM-A) (CD antigen CD321)	0,5018	0,00004
Q9R0E1	Plod3	Multifunctional procollagen lysine hydroxylase and glycosyltransferase LH3	0,5021	0,00000
P21619	Lmnb2	Lamin-B2	0,5070	0,00000
Q4FJS7	F10	F10 protein	0,5123	0,00015
Q4FJR9	Isg15	G1p2 protein (ISG15 ubiquitin-like modifier)	0,5130	0,00000
O88668	Creg1	Cellular repressor of E1A-stimulated genes 1	0,5283	0,00001
D3YXQ6	Psg16	Pregnancy-specific glycoprotein 16 (Fragment)	0,5309	0,00066
A0A0R4J1N3	Apoc3	Apolipoprotein C-III	0,5319	0,00000
A0A0R4J039	Hrg	Histidine-rich glycoprotein	0,5381	0,00001
Q3TL44	Nlrx1	NLR family member X1	0,5412	0,00000
O35258	Prl8a2	Decidual/trophoblast prolactin-related protein	0,5417	0,00003
A0A2I3BPR3	Gm49394	Predicted gene	0,5475	0,00002
P28665	Mug1	Murinoglobulin-1	0,5508	0,00000
Q3UEK1	Mbl2	Mannose-binding protein C	0,5512	0,00000
Q9ET52	Cts6	Cathepsin-6	0,5519	0,00003
A0A0M6L0X0	Prl8a6	Growth hormone d13	0,5524	0,00001
Q3URC9	Ace2	Angiotensin-converting enzyme	0,5539	0,00000
Q5NCU4	Sparc	SPARC	0,5551	0,00007
Q501P1	Fbln7	Fibulin-7	0,5557	0,00000
A0A0M6L0N6	Prl8a9	Growth hormone d15	0,5769	0,00014
Q9D236	Htra3	Serine protease HTRA3	0,5864	0,00152

Q52L64	Igkv8-30	ENSMUSG00000076577 protein	0,5879	0,00002
Q3UKR1	Dcn	Decorin (Bone proteoglycan II)	0,5937	0,00079
Q9D7N9	Apmap	Adipocyte plasma membrane-associated protein	0,5964	0,00003
P57759	Erp29	Endoplasmic reticulum resident protein 29	0,6126	0,00000
Q3KQQ4	Serpina1b	Serpina1a protein	0,6373	0,00001
Q07797	Lgals3bp	Galectin-3-binding protein	0,6409	0,00000
Q8R5L1	C1qbp	Complement component 1 Q subcomponent-binding protein; mitochondrial	0,6522	0,00002
B2RPR7	Itga2b	Integrin alpha 2b	0,6540	0,00003
Q9R118	Htra1	Serine protease HTRA1	0,6564	0,00014
Q60962	Psg16	Brain carcinoembryonic antigen	0,6704	0,00004
P43275	H1-1	Histone H1.1	0,6775	0,00000
P97450	Atp5pf	ATP synthase-coupling factor 6; mitochondrial	0,6837	0,00003
Q9DBH5	Lman2	Vesicular integral-membrane protein VIP36	0,6878	0,00000
A0A0F7IQ06	Itgav	Alpha v integrin	0,6992	0,00002
P67778	Phb	Prohibitin	0,7014	0,00000
Q80YP5	Itga5	Integrin alpha 5	0,7053	0,00000
Q91WQ0	Serpina6	Serine (Or cysteine) peptidase inhibitor; clade A; member 6	0,7155	0,00000
Q3TWM9	Psap	Prosaposin (Fragment)	0,7230	0,00001
P05202	Got2	Aspartate aminotransferase; mitochondrial	0,7451	0,00000
