# Supplementary figures and images for: Mice Placental ECM Components May Provide A Three-Dimensional Placental Microenvironment
Source: Bioengineering (Basel). 2022 Dec 22;10(1):16. doi: 10.3390/bioengineering10010016 (PMC9855196; doi:10.3390/bioengineering10010016)

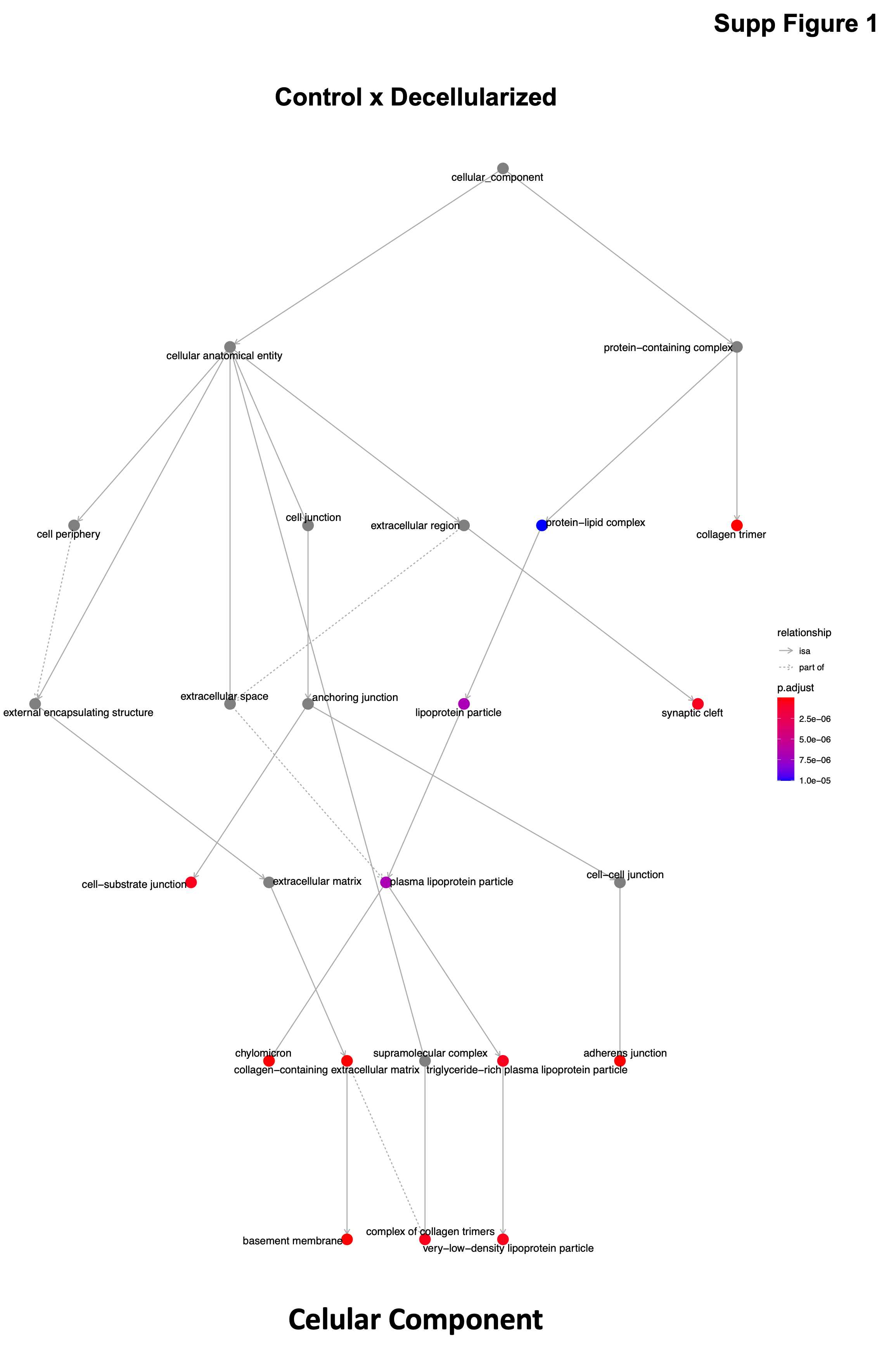

Supplement: Supplementary file 1 [file bioengineering-10-00016-s001.zip › Supp Figure 01.png]

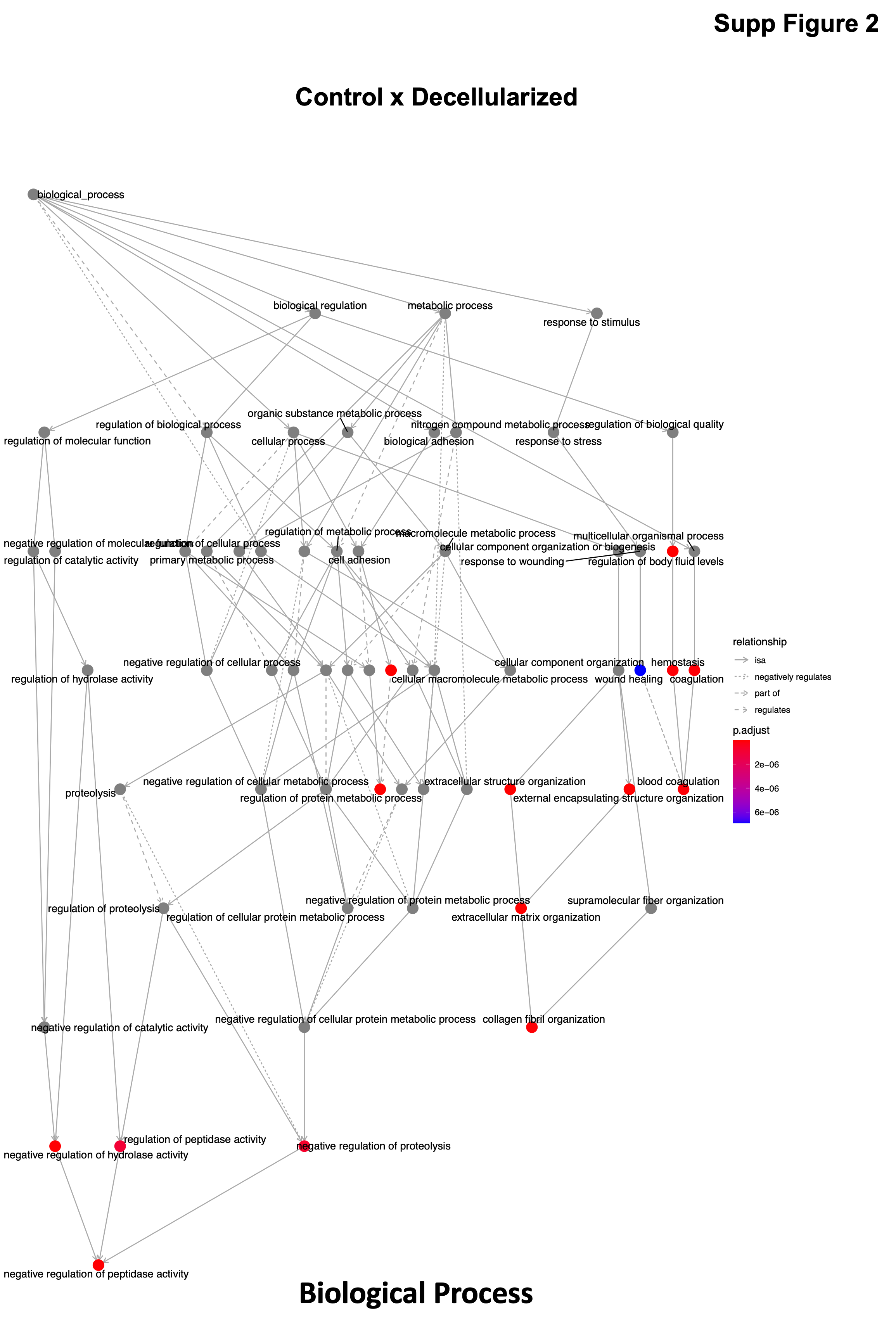

Supplement: Supplementary file 1 [file bioengineering-10-00016-s001.zip › Supp Figure 02.png]

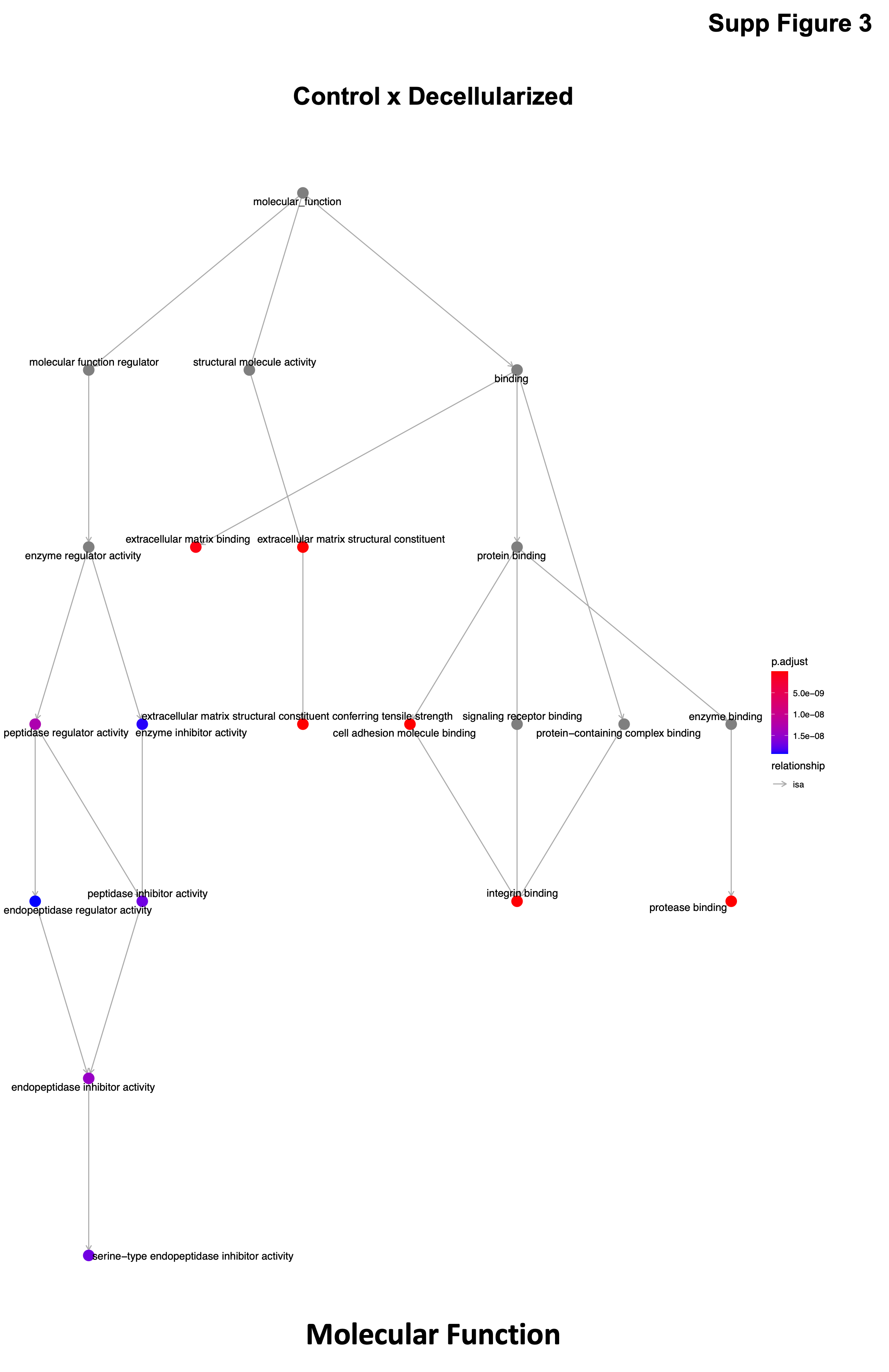

Supplement: Supplementary file 1 [file bioengineering-10-00016-s001.zip › Supp Figure 03.png]

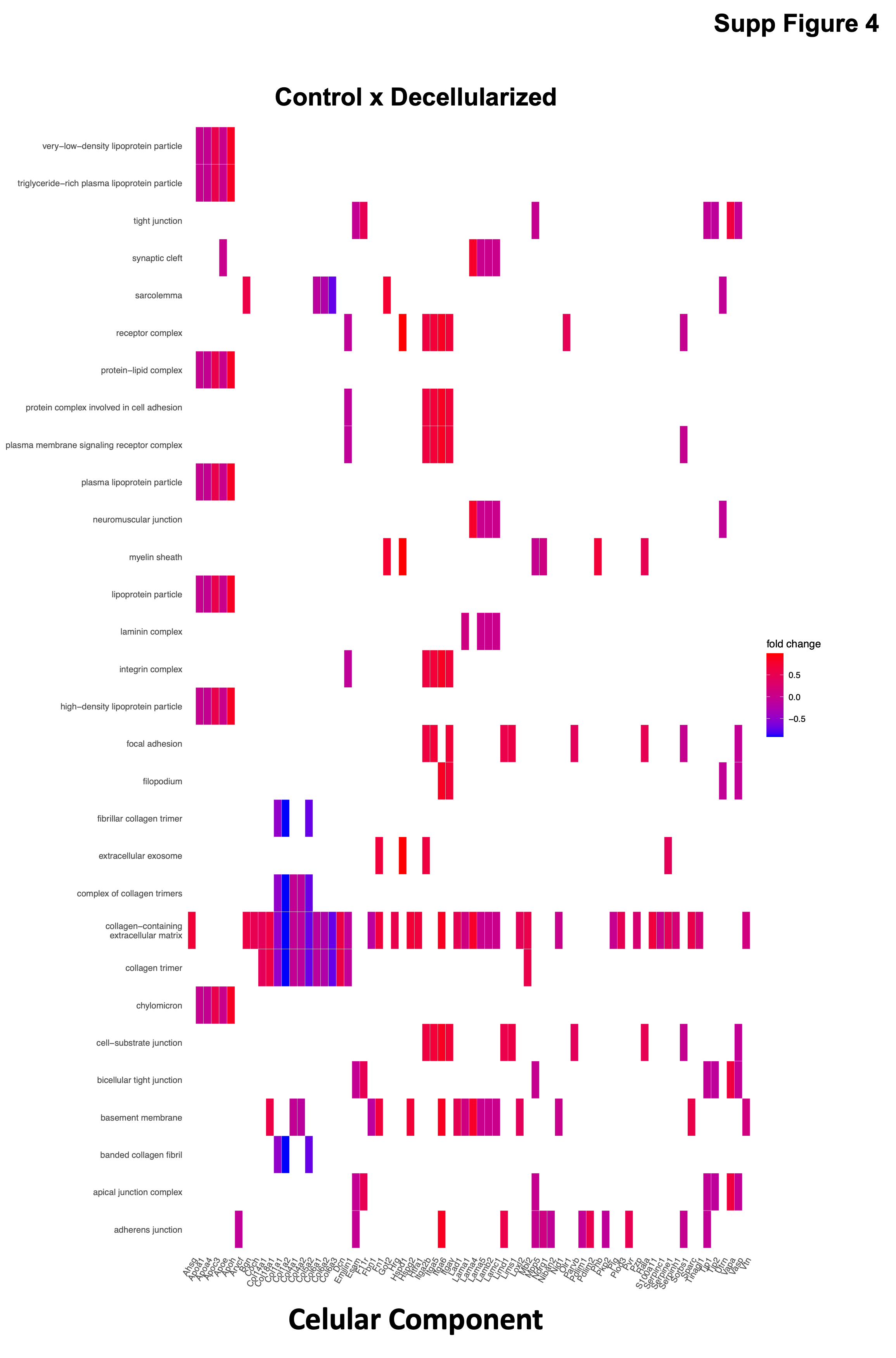

Supplement: Supplementary file 1 [file bioengineering-10-00016-s001.zip › Supp Figure 04.png]

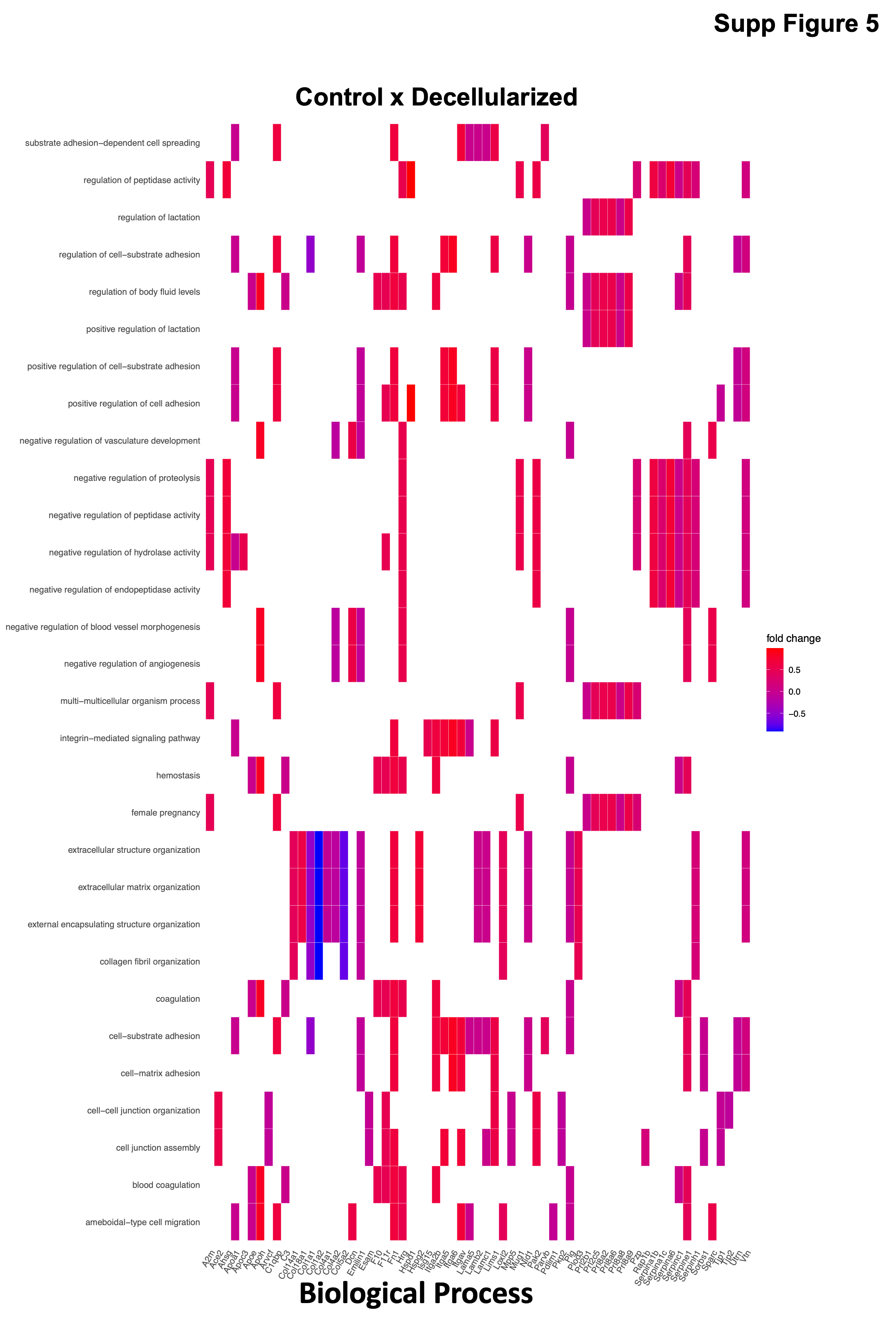

Supplement: Supplementary file 1 [file bioengineering-10-00016-s001.zip › Supp Figure 05.png]

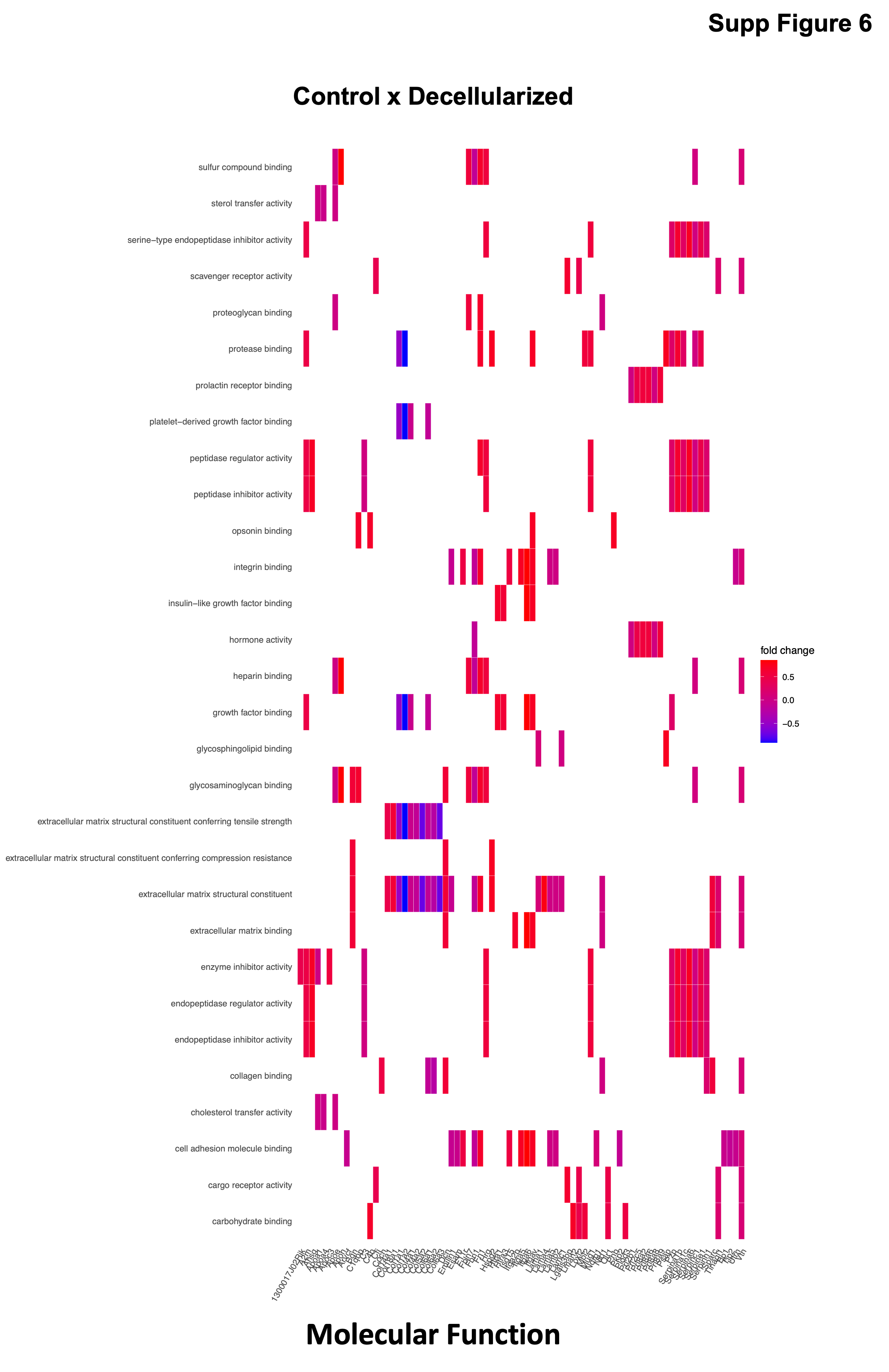

Supplement: Supplementary file 1 [file bioengineering-10-00016-s001.zip › Supp Figure 06.png]

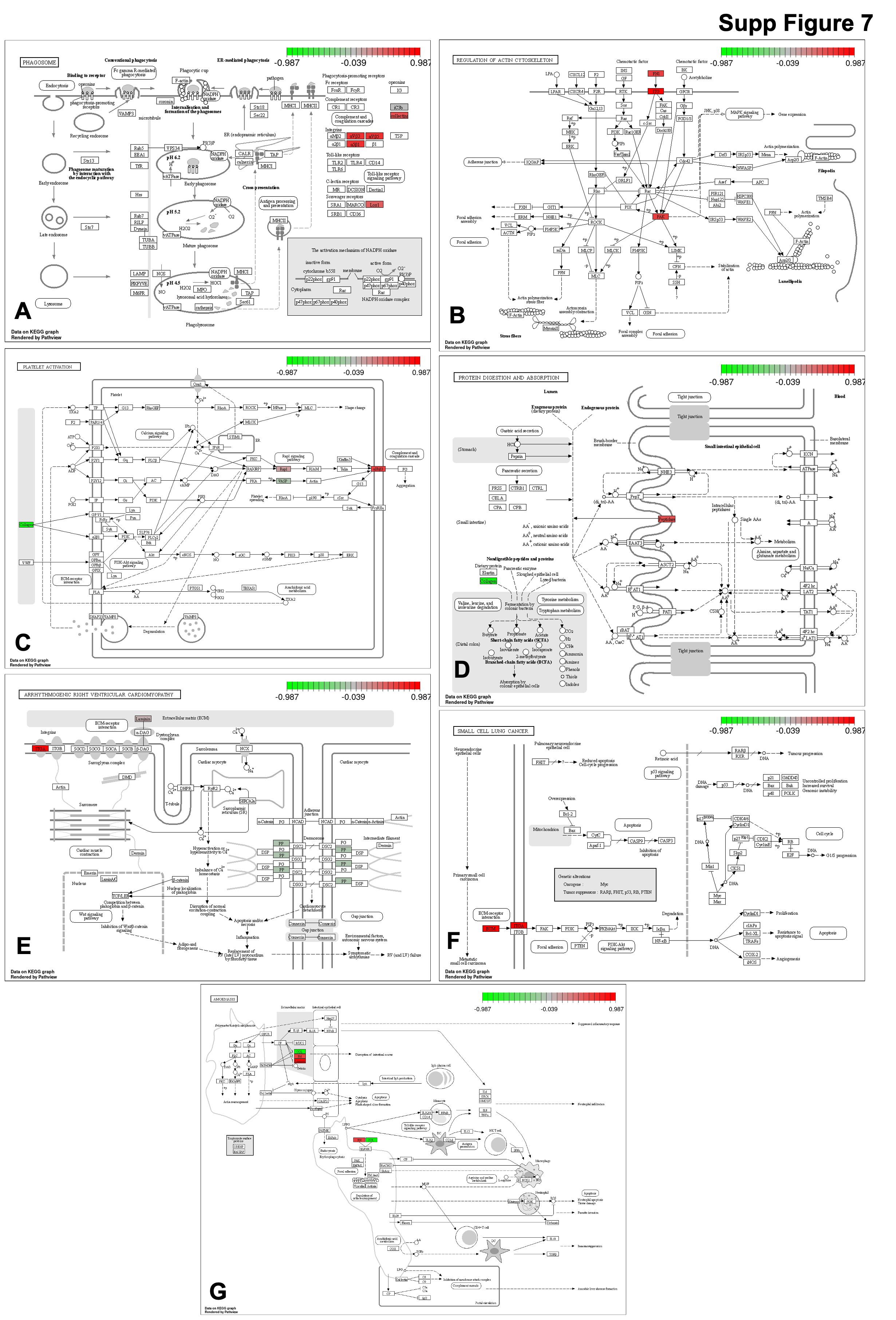

Supplement: Supplementary file 1 [file bioengineering-10-00016-s001.zip › Supp Figure 07a.png]

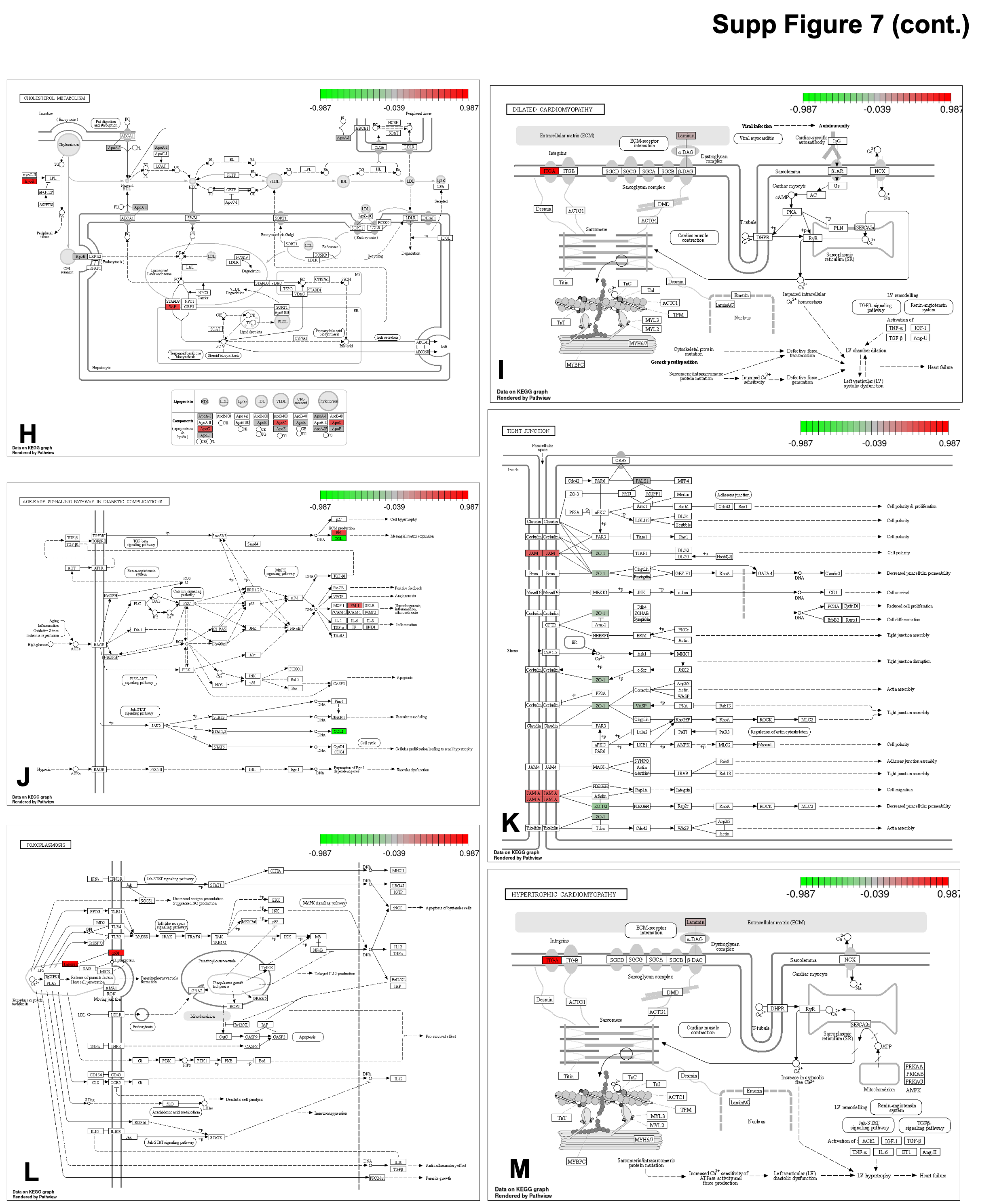

Supplement: Supplementary file 1 [file bioengineering-10-00016-s001.zip › Supp Figure 07b.png]

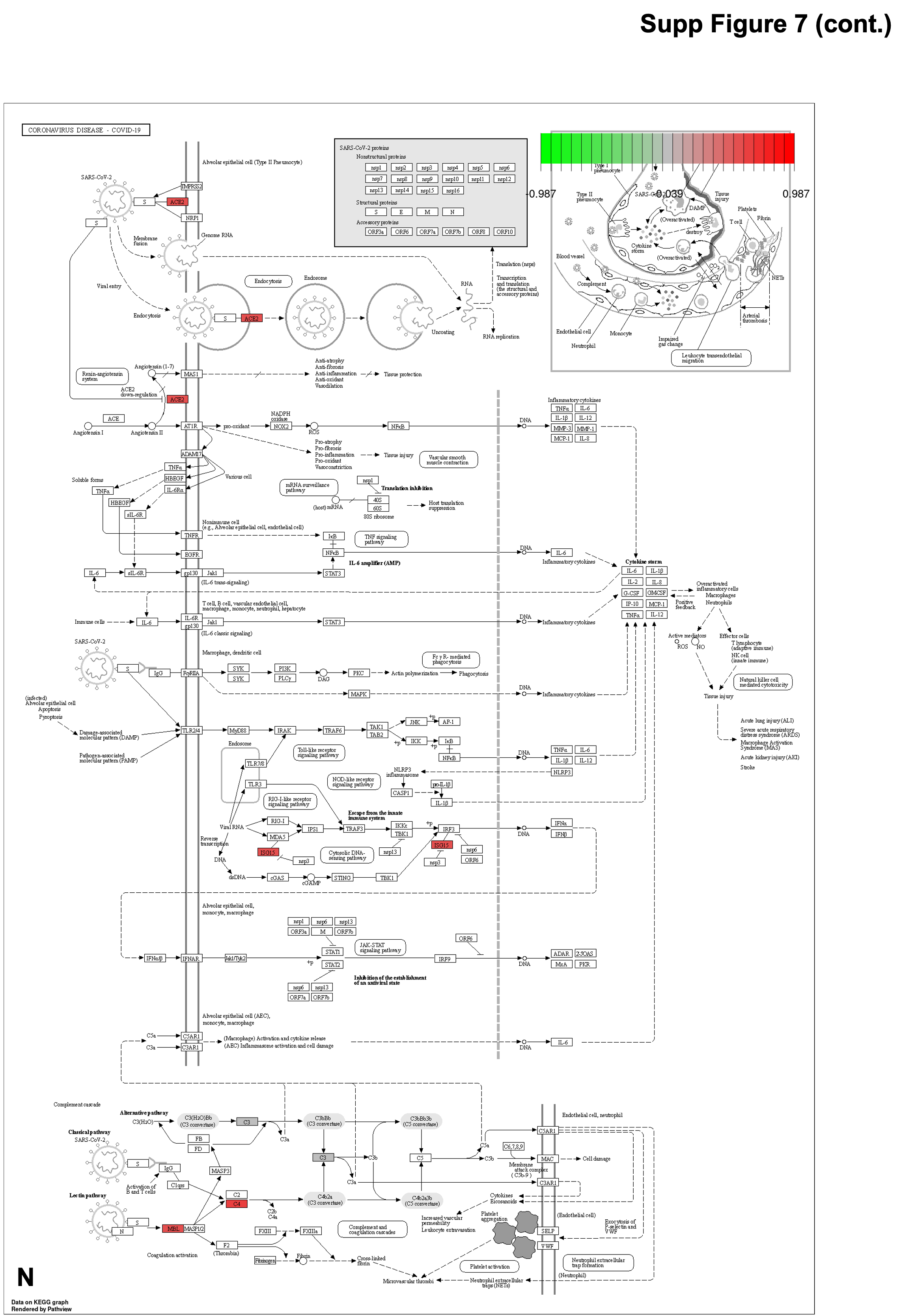

Supplement: Supplementary file 1 [file bioengineering-10-00016-s001.zip › Supp Figure 07c.png]
